# Effects of a Nursing Intervention Based on a Solution-Focused Approach on Renal Transplant Recipients' Anxiety, Depression, and Quality of Life

**DOI:** 10.1155/2023/4920799

**Published:** 2023-11-09

**Authors:** Shimin Hu, Chunyu Yuan, Qingzhu Lu, Xiaoyong Yan, Yan Huang, Minzhu Chen, Yong Liu, Zhouke Tan, Mingtao Quan

**Affiliations:** ^1^Department of Nephropathy, Affiliated Hospital of Zunyi Medical University, Zunyi, China; ^2^Nursing Department, Affiliated Hospital of Zunyi Medical University, Zunyi, China; ^3^Nursing School of Zunyi Medical University, Zunyi, China

## Abstract

**Objective:**

To explore the effects of a nursing intervention based on a solution-focused approach on improving renal transplant recipients' anxiety, depression, and quality of life.

**Methods:**

A total of 75 eligible recipients who underwent renal transplantation were recruited and randomly divided into intervention and control groups. The renal transplantation recipients in the intervention group received nursing intervention based on a solution-focused approach (SFA) developed by the research group. The SFA intervention included the following five stages: describing the problem, developing well-formed goals, exploring for exceptions, end of session feedback, and evaluating progress. Additional methods, such as empowerment, miracle questions, and scale mark questions, were included. The intervention began after informed consent, and baseline data were collected at admission, with each participant receiving five interventions lasting 30–60 minutes. The featured methods and five stages of the SFA could be interspersed and used repeatedly. The follow-up was performed at one, three, and six months postoperation. The control group received the usual care of comparable length and follow-up contact. The anxiety, depression, and quality of life of renal transplant recipients were measured and recorded using the Hospital Anxiety and Depression Scale (HADS) and the Quality of Life Scale for Patients of Renal Transplantation (QOL-RT). A trained research nurse collected all the baseline and follow-up data.

**Results:**

The baseline information of the patients, such as gender, age, BMI, endogenous creatinine clearance, anxiety, and depression, was similar between the two groups (*P* > 0.05). The total scores of HADS (A), HADS (D), and total HADS in both groups showed a downward trend. The intervention group exhibited significantly lower HADS (A) (4.21 ± 1.85) and total HADS scores (7.81 ± 3.31) one month after surgery than the control group in the same period (5.50 ± 2.44 and 9.85 ± 4.19, respectively; *P* < 0.05). Fewer people in the intervention group had a HADS (A) score ≥8 than those in the control group at one month (*P* < 0.05). Depression in the intervention group was significantly lower than that in the control group at three and six months (*P* < 0.05). The total QOL-RT scores of the intervention group at one month (126.54 ± 9.62), three months (137.02 ± 7.69), and six months (144.89 ± 7.53) were higher than those of the control group (119.50 ± 11.58, 128.8 ± 10.80, and 138.61 ± 9.09, respectively; *P* < 0.05). Furthermore, the scores of the physiological function dimensions and treatment dimensions of the QOL-RT in the intervention group were higher than those in the control group at one, three, and six months after the intervention (*P* < 0.05). The scores of the social function dimension in the intervention group were higher than those in the control group at three and six months after the intervention (*P* < 0.05).

**Conclusion:**

Nursing intervention based on the SFA improved anxiety and depression among renal transplant recipients, thereby improving their quality of life.

## 1. Introduction

Renal transplant recipients are prone to anxiety and depression, which are the most common mental health problems, as they experience long-term chronic renal disease, surgical stress trauma, and social and economic pressure [1–3]. Research shows that anxiety and depression have a serious impact on the quality of life of renal transplant recipients and increase the incidence of complications and even lead to death [4–6]. Taking effective measures to help renal transplant recipients reduce anxiety and depression could improve the incidence of adverse outcomes and their quality of life. Previous studies have examined this issue. Hogan and Silverman [7] improved the emotions of transplant patients through onsite music therapy courses combined with coping strategy dialogues. Reilly-Spong et al. [8] conducted mindfulness stress reduction training on patients who were about to undergo renal transplantation, and the results showed improvements in depression and anxiety. Han [9] used attributive therapy to intervene in renal transplantation patients, and the negative emotions of the patients in the early postoperative period were improved. However, most of these studies were led by doctors or nurses and did not stimulate patients' active participation. Van Hooft et al. [10] noted that achieving good results is difficult if the nursing intervention does not encourage the patients to participate, arouse the confidence of the patients to change their behaviour, or show patients positive results immediately following their efforts. Therefore, the key to improving the negative emotions and behaviours of renal transplant recipients is to guide them to participate in treatment, help them build confidence in overcoming the disease, and improve their ability to solve problems.

The solution-focused approach (SFA) is a clinical intervention model proposed by Shaza [11] in the late 1970s and developed under the background of positive psychology. The SFA model includes the following five stages: describing the problem, developing well-formed goals, exploring exceptions, end of session feedback, and evaluating progress. The SFA emphasises patient-centred care and does not pursue the causes of past failures and problems, focusing on exploring patients' advantages and strengths through psychotherapy techniques, such as listening, miracle questions, empowerment, and solution construction. This approach aims to increase positive expectancies and emotions, such as hope and optimism, and actively identify solutions to patients' problems. Practitioners work with patients by expressing respect for and trust in them and encouraging active participation in treatment and care rather than passive acceptance. The SFA helps patients build self-confidence in overcoming their disease. Self-confidence is a valuable personal asset. Confidence is an important part of ability, and psychology advocates that “believing in yourself” is the key to personal success [12, 13].

The SFA focuses on the positive qualities of patients, promotes positive coping behaviours, and effectively improves patients' self-confidence and problem-solving abilities [14]. The SFA has been used worldwide, and its application effect has been effectively demonstrated in the clinical environment, [15] especially for improving anxiety and depression. For instance, the SFA was effective in improving anxiety and depression in cases of chronic diseases and among adolescent patients [16–18]. However, reports on the application of the SFA in the field of renal transplantation are scarce. Renal transplant patients usually suffer from chronic renal disease; thus, we hypothesised that the SFA would be equally effective in patients with renal transplants. Therefore, this study applied the SFA to the nursing care of renal transplant recipients and examined its effects on their anxiety, depression, and quality of life to provide new ideas for nursing intervention in renal transplantation.

## 2. Materials and Methods

### 2.1. Study Design

This study was a randomised controlled trial. The participants were numbered according to the order of admission, and a third party who did not participate in the later intervention used the SPSS random number generator to generate random numbers. The participants were randomly divided into two groups following a 1 : 1 ratio. A single-blind method was used in this study, and the outcome evaluators were not aware of the grouping and allocation scheme. This study was approved by the Medical Ethics Committee of the Affiliated Hospital of Zunyi Medical University. The batch number for the ethical review is KLLY-2020-013.

### 2.2. Participants

The quality of life of renal transplant recipients was used as the outcome index. After combining the intervention results of two studies, the average score of the quality of life after intervention was 139.01 [19, 20]. The standard deviation was 10.91 (*α* = 0.05 and *β* = 0.10). The two groups of samples were the same (*n*1 = *n*2). According to the calculation of PASS software, approximately 25 cases were needed in each group. The corrected sample size was calculated by considering the loss rate of 20%. The final number of samples needed in this experiment was 60, with 30 cases in the control group and 30 cases in the intervention group. The calculation formula was *n*1 = *n*2 = 2 ([*t*_*α*_ + *t*_*β*_]^2^*s*/*δ*)^2^. The inclusion criteria were as follows: (1) recipients of an allograft renal transplantation in accordance with the Regulations on Human Organ Donation, (2) age ≥18 years, and (3) patients with communication and reading comprehension abilities. The exclusion criteria were as follows: (1) patients who were not first-time recipients of renal allotransplantation; (2) patients with multiple organ transplants; (3) patients with serious, life-threatening complications after surgery; and (4) patients with previous mental illness and those who were taking antidepressants. The elimination and shedding criteria were as follows: (1) those who withdrew participation during the study; (2) study interruptions due to changes in illness; and (3) patients with whom communication was lost due to a change in contact information during the course of the study.

### 2.3. Intervention and Control Condition

#### 2.3.1. Intervention Condition

A preliminary SFA intervention scheme for renal transplant recipients was constructed based on previous study results [11, 21–23] and clinical practice. The first draft of the intervention plan was revised and improved through consultation with ten experts (two nephrology specialists, two renal transplant doctor specialists, two renal transplant care specialists, one psychologist, one critical care medicine specialist, one rehabilitation specialist, and one nutritionist). Finally, we conducted a pretrial in the clinic to test the scientific quality and feasibility of the program. Based on the problems identified during the pretrial, we revised the intervention plan to form the final SFA care program.

We established a research group with eight team members (one renal transplant doctor, one renal transplant nurse, one intensive care nurse, one psychological counsellor, one dietitian, one rehabilitation nurse, and two research nurses). The renal transplant nurse had completed a psychology course and a course on the SFA theory and understood the intervention method and the process of the SFA. The renal transplant nurse had worked in the renal transplant ward for more than ten years and had a good understanding of renal transplant recipients as well as good clinical and communication skills. The renal transplant nurse was responsible for training the other members of the research group to ensure that all SFA implementers could use the SFA methods and processes. The renal transplant nurse delivered the intervention program. Nutrition rehabilitation and psychological problems encountered during program implementation were guided and assisted by trained dietitians, rehabilitation nurses, and psychological counsellors in accordance with the SFA process. The renal transplant doctor screened patients strictly according to the inclusion and exclusion criteria. One research nurse conducted the randomisation and placed each randomised number in a sealed opaque envelope. Blindness for patients could not be achieved, as the intervention program was only available to patients in the intervention group. Nevertheless, data collection was completed by another research nurse who did not know the grouping of random subjects. The specific implementation steps of the research and the division of labour among team members are shown in Table 1.

Participants in the intervention group underwent a nursing intervention based on the SFA. The intervention time was from the collection of the baseline data to the discharge of the recipient. An SFA-based nursing intervention was given one day before renal transplantation surgery, one day after the surgery, three days after the surgery, seven days after the surgery, and before discharge. Intervention sessions lasted 30–60 minutes, with a total of five sessions. The intervention was performed in the renal transplant ward or in a separate room in the department. Individual face-to-face interventions were adopted. The specific intervention stages and contents were as follows.

1. Describing the problem. This included (a) guiding renal transplant recipients to express their true feelings, needs, and social relations by listening, asking, and chatting; (b) understanding their existing problems, available resources, and psychological readiness; and (c) establishing a trusting relationship with recipients and their families. At this preoperative stage, we addressed the patients' doubts and helped them fully prepare for their operation by distributing relevant materials of preoperative renal transplantation health education.
2. Developing well-formed goals. Doctors and nurses, psychological counsellors, dietitians, family members, and renal transplant recipients were invited to discuss their current difficulties and coping strategies. The researcher distributed the relevant materials related to health education of renal transplantation and worked with renal transplant recipients to help them set realistic expectations, goals, and action plans. The patients were motivated to make continuous efforts to achieve the goals. For instance, renal transplant recipients often have lower back pain, wound pain, and poor sleep after surgery. Furthermore, due to concerns about the urine volume and creatinine value, patients may experience tension, anxiety, and antipathy. According to the specific situation, the researcher made an appointment with the recipient to discuss the causes and consequences of pain, sleep disorders, and anxiety and set phased goals with the recipient. For instance, the daily pain score could be reduced by two points. The time from going to bed to going to sleep could be shortened by more than one hour. The recipients should feel less nervous than before if the urine volume changes. At this stage, the researcher provided timely and personalised intervention to the patients to address existing problems. This included having patients watch their favourite programs, distributing materials, and encouraging recipients to enrich their daily life by chatting with relatives on the phone to divert attention, self-study, writing transplant diaries, and recording self-monitoring indicators.
3. Exploring exceptions. The miracle questions based on the SFA-exception inquiry method guided the recipient to recall which problems were solved and their past or current efforts and experiences that provided a valuable reference for follow-up rehabilitation. During this process, the focus characteristic inquiry method was used to explore the exceptions repeatedly to fully stimulate the potential of renal transplant recipients. Questions included “what were your previous achievements?,” “under what circumstances have you achieved success or progress?,” “you slept better last night than the day before. How did you do it?” “your activity is very good, so your excretions are quicker than expected; you are doing great! It is great that you got out of bed to measure your weight today. How did you do this?” The miracle questions are a method of inquiry with focus features. By helping the recipient constantly explore exceptions in the process of intervention, the interventionist can help the recipient realise their own potential and resources for solving problems and learn to tap into their own abilities and resources, thereby improving their confidence in achieving their goals.
4. Positive feedback. This included encouraging the achievement of goals in an affirming and timely manner and stimulating the potential of the recipient to take the initiative to solve the problem. For unmet goals, stage 2 was revisited. For instance, the patients were told “all the oral medicines you took today are correct. That is great!” Patients who wanted to quickly return to regular life and failed to achieve their expected goals were told “recovery after transplantation is a comprehensive and gradual process, and there should be no haste.” Moreover, they could return to stage 2 and adjust their goals to make them more feasible and specific (e.g., “set a goal that if you can exercise properly at your bedside tomorrow, you can go to the bathroom the day after tomorrow”).
5. Evaluating progress. The method of scaled questioning was used to allow the recipients to independently evaluate the realisation of their goals. Furthermore, experiences were summarised, and the construction of new goals was guided, with a continued strive for greater progress. At this stage, through scale evaluation, recipients could intuitively see their progress and enhance their ability and confidence to gradually solve problems (e.g., “use a scale of 1–10 to rate your emotional management. You rated 1 on the second day of the operation. How many points do you think it is get now?” “Use a score of 1–10 to score your sleep. Yesterday, you rated your sleep as 2. How many points do you think it is today?”).

The five stages of the intervention process and the characteristic methods of each stage can be interspersed and cycled.

#### 2.3.2. Control Condition

The participants in the control group did not receive the SFA intervention program, while other nursing measures were the same. Routine and psychological nursing care for renal transplantation was provided to the control group. During hospitalisation, doctors and nurses in the renal transplant care unit conducted nursing according to the routine of admission nursing, preoperative preparation, postoperative nursing, and discharge guidance for renal transplant recipients.

### 2.4. Fidelity of the Intervention Protocol

The intervention program was reliable and feasible, as its construction was based on a literature review and previous survey results, revised through expert meetings and rigorously tested. Team members conducting the intervention received training and were required to follow the program protocol strictly. We formed a research team and were able to communicate and deal with problems that arose during program implementation in a timely manner.

Furthermore, we took measures to avoid contamination of different groups. The intervention was conducted in a separate room or studio in the department, and the participants did not know their grouping. To ensure the accuracy of data collection, the outcome evaluators were trained and proficient in the use of each scale. During data collection, uniform instructions were used to fill in the questionnaires and the questionnaires were checked immediately after they were returned to ensure the integrity of the data. Moreover, the outcome assessors were blinded to the grouping and the protocol.

### 2.5. Outcomes

Before the intervention and one, three, and six months after the intervention, the general data sheet, the Hospital Anxiety and Depression Scale (HADS), and the Quality of Life Scale for Patients of Renal Transplantation (QOL-RT) were used to collect data.

#### 2.5.1. General Information Sheet

The general information sheet was designed by the researchers according to the purpose of the study and included the participants' general data, such as gender, age, marital status, and education level, and disease-related data, such as the preoperative dialysis mode and dialysis time.

#### 2.5.2. Hospital Anxiety and Depression Scale

The HADS is commonly used to measure the psychological state of anxiety and depression [24]. The scale is divided into anxiety (HADS (A)) and depression (HADS (D)) subscales, with seven items in each subscale, for a total of 14. Items are graded from 0 to 3. Subscale scores are categorised as follows: 0–7 (asymptomatic), 8–10 (possible existence of symptoms), and 11–21 (existence of symptoms). The HADS has good reliability [25]. The Cronbach's alpha values of each subscale were >0.8. The internal correlation coefficients (ICC) of HADS, HADS (A), and HADS (D) were greater than 0.9, indicating that the scale was stable.

#### 2.5.3. Quality of Life Scale for Patients of Renal Transplantation

The 34-item QOL-RT consists of the following five dimensions: (1) physiological function dimension, (2) psychological function dimension, (3) social function dimension, (4) treatment dimension, and (5) overall quality of life dimension. The scale evaluates the quality of life of renal transplant recipients from physical, psychological, social, and therapeutic aspects. Items are rated on a five-point Likert scale (1 = never and 5 = always). The score ranges from 34 to 170. Higher scores indicate better quality of life. Scores of <102, 102–136, and >136 are considered poor, medium, and good, respectively. The content validity index (CVI) and Cronbach's alpha coefficient of the scale were 0.96 and 0.86, respectively. The scale has been extensively used and proven to have high reliability and validity [24].

### 2.6. Statistical Analysis

In this study, Microsoft Excel 2016 was used for double data entry. After checking and comparing, a database was established. Data were imported into SPSS 18.0 (IBM Corp., Armonk, NY, USA) for statistical analysis, and *P* < 0.05 was considered statistically significant. Count data were expressed as frequencies and percentages and measurement data as means and standard deviations. Repeated measurement analysis of variance (ANOVA) was used to compare the measurement data at multiple time points. The F interaction effect was group × time, the F intergroup effect was grouping (intervention group and control group), and the intragroup effect was at each time point (baseline and one, three, and six months after surgery). If the repeated measurement data did not conform to the normal distribution or was grade data or a binary variable, the generalised estimating equations were used for statistical analysis. *T*-tests were used to compare the difference between the two averages. For measurement data that were in accordance with the normal distribution, we used a two independent samples *t*-test to analyse the difference between the two groups. Measurement and grade data that did not conform to the positive distribution and the binary classification data were statistically analysed by the rank sum or chi-square tests.

## 3. Results

### 3.1. Participants' Flow through the Trial

**Figure 1:**
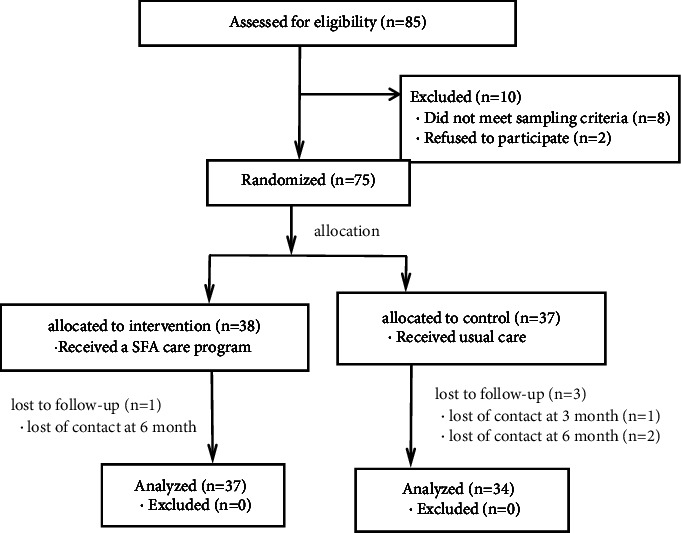
Flowchart showing patient selection, randomization, follow-up, and analysis.

From September 2020 to June 2021, researchers screened 85 renal transplant recipients and excluded ten (of which eight were second-time renal transplants and two refused to participate). Finally, 75 participants were randomly assigned (38 in the intervention group and 37 in the control group). All patients' baseline data were obtained at admission. The follow-up data were collected at one, three, and six months postoperation. The baseline and follow-up data were completed by a trained research nurse who did not know about the grouping and did not participate in the intervention. During the study period, one participant dropped out of the intervention group (2.6; they could not be contacted six months after operation) and three in the control group (8.1%, two failed to be reexamined at six months and one did not follow-up at three months). The total drop-out rate was 10.7%.  Figure 1 shows the process of patient selection, randomisation, follow-up, and analysis.

### 3.2. Comparison of General Demographic and Disease Data between Groups

Seventy-one renal transplant recipients finished the study, including 45 males (63.4%) and 26 females (36.6%). Of them, 55 (77.5%), 14 (19.7%), and 2 (2.8%) were on haemodialysis, peritoneal dialysis, and not dialysed, respectively. No significant difference was found in comparing the baseline data (general demography data and disease data) of the two groups of renal transplant recipients (*P* > 0.05; Table 2).

### 3.3. Comparison of Anxiety and Depression Scores between Groups before and after the Intervention

**Figure 2:**
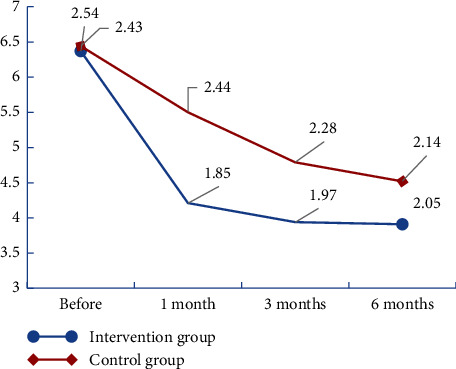
Change trend of HADS (A) scores in the two groups of renal transplant recipients.

**Figure 3:**
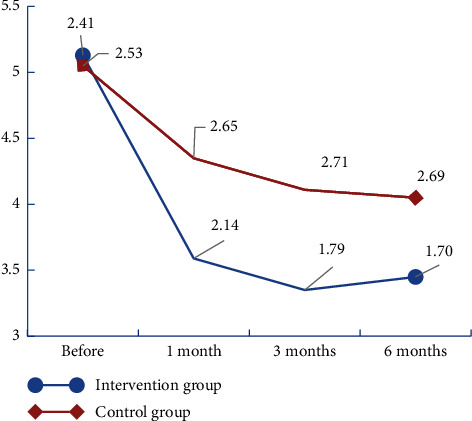
Change trend of HADS (D) scores in the two groups of renal transplant recipients.

**Figure 4:**
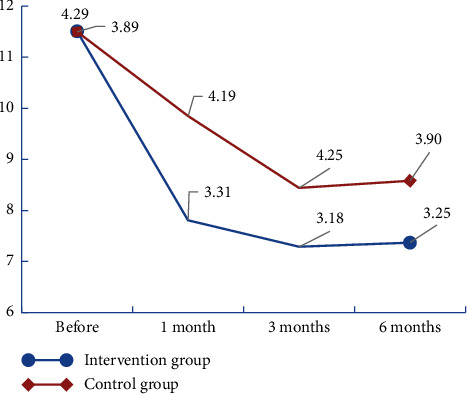
Change trend of HADS scores in the two groups of renal transplant recipients.

The results showed that the total scores of HADS (A), HADS (D), and HADS decreased in both groups after the intervention (Figures 2–4). We found that there was no significant group × time interaction. However, HADS (A) and total HADS one month after the intervention were significantly lower in the intervention group (4.21 ± 1.85 and 7.81 ± 3.31, respectively) than in the control group (5.50 ± 2.44 and 9.85 ± 4.19, respectively) (*P* < 0.05; Table 3). The results of the generalised estimating equations' analysis showed that the number of patients with anxiety scores ≥8 at one month was statistically significantly less in the intervention group than in the control group (*t* = −0.21 and *P* = 0.012 < 0.05). At three months, the number of patients with depression scores ≥8 was statistically significantly less in the intervention group than in the control group (*t* = −0.24 and *P* = 0.003 < 0.05). At six months, the number of people with a HADS (D) score ≥8 was statistically significantly less in the intervention group than in the control group (*t* = −0.18 and *P* = 0.016 < 0.; Table 4).

### 3.4. Comparison of Quality of Life Scores between Groups

The results showed that total QOL-RT scores and scores in each dimension increased. Physiological function, treatment, and total QOL-RT in the intervention group were significantly higher than those in the control group at one, three, and six months after the intervention. The score of the social function dimension in the intervention group was significantly higher than that in the control group at three and six months after the intervention (*P* < 0.05). Three months after the intervention, the overall quality of life score in the intervention group was significantly higher than that in the control group (*P* < 0.05; Table 5).

## 4. Discussion

### 4.1. Nursing Intervention Based on a Solution-Focused Model Relieved Anxiety and Depression in Renal Transplant Recipients

Anxiety and depression are the most common mental health problems among renal transplant recipients. When infections, drug side-effects, and other complications occur, the anxiety of renal transplant recipients significantly increases [26]. If the recipients realise that transplantation cannot restore their lives to the state before renal disease or do not engage in corresponding coping strategies in time, they are extremely prone to anxiety and depression [27]. Anxiety and depression are risk factors for poor self-management ability, poor compliance, physical activity disorders, disease progression, and adverse outcomes in renal transplant recipients [28, 29].

This study implemented an SFA-based nursing intervention, which reduced anxiety and depression among renal transplant recipients. The anxiety and depression scores of the two groups were higher before transplantation, which may be related to the fear of surgical risks and postoperative complications. Previous studies have found that more than half of the patients had anxiety before the operation [30, 31]. Therefore, in this study, the researchers administered an SFA-based nursing intervention according to the results of the baseline measurement of anxiety and depression in renal transplant recipients. Based on the causes of anxiety and depression of the recipients and the existing problems, it is necessary to give individualised guidance so that they can correctly understand and face the transplantation operation and implement targeted coping strategies. After the intervention, the results showed that the anxiety and depression scores in both groups decreased postoperatively, which was consistent with the results of Tavallaii and Lankarani [32]. One month after the intervention, HADS (A) and HADS scores in the intervention group were lower than those in the control group. One month after the intervention, fewer patients had a HADS (A) score ≥8 in the intervention group than in the control group; three months after the intervention, fewer patients had a HADS (D) score ≥8 in the intervention group than in the control group; and six months after the intervention, fewer patients had a HADS (D) score ≥8 score in the intervention group than in the control group. Therefore, the nursing intervention based on an SFA effectively reduced the incidence of anxiety and depression in renal transplant recipients, which may be related to the better psychological preparation of the recipients after the preoperative SFA nursing intervention. Moreover, in SFA-based nursing interventions, our focus is to fully exploit the resources and potential of renal transplant recipients and emphasise their personal advantages rather than deal with their shortcomings and experiences of failure [33]. We encouraged self-reflection and active expression by the renal transplant recipients, allowing them to put forward small but practical goals according to their own condition at each stage. In addition, we used methods such as the “exceptional inquiry method,” “positive feedback method,” and “scaled questioning” to enable renal transplant recipients to see their progress in a timely and accurate manner, realise the hope brought by positive progress and benefits, discover the feeling of success, rebuild their self-confidence by continuously guiding them to accumulate successful experiences, and be relieved of their anxiety and depression. As psychology advocates, “believing in yourself” is the key to personal success [12]. In the process of SFA nursing, we helped renal transplant recipients better accept and affirm themselves and cope with difficulties with a positive and optimistic emotional state.

As evidenced by numerous studies, the use of the SFA as a strength-based treatment in clinical practice is effective [34, 35]. Our study revealed differences in HADS (A) and HADS scores between the two groups of recipients one month after the intervention. However, there was no significant group × time interaction. Furthermore, there were no significant differences in HADS (A) and HADS scores between the two groups three and six months after the intervention. Previous research indicates that the SFA appears particularly effective as an early intervention, when problems first appear [36]. However, the long-term effects remain to be confirmed in future research.

### 4.2. Nursing Intervention Based on a Solution-Focused Model Improved the Quality of Life of Renal Transplant Recipients

The main purpose of renal transplantation is to restore physical and mental health and improve the quality of life of the recipient [37]. The results of this study showed that the scores of the QOL-RT physiological function dimension, psychological function dimension, social function dimension, treatment dimension, and overall quality of life dimension in the intervention group were higher than those in the control group after intervention. The total score of QOL-RT in the intervention group was also higher than that in the control group. This highlights that an SFA-based nursing intervention can improve the physiological, psychological, and social function of renal transplant recipients and improve their quality of life.

During the implementation of an SFA-based nursing intervention, we strengthened communication with renal transplant recipients and their families by describing problems, jointly setting goals, sharing decisions, and establishing supportive relationships for them [33, 38]. SFA-based nursing aims to identify the positive behaviour and successful experience of renal transplant recipients and maximise their potential strengths, which is very effective in helping recipients actively participate in decision-making [35]. Furthermore, in the description of the problem, we focused on the needs, problems, and weaknesses of renal transplant recipients. We focused on the resources and advantages that renal transplant recipients already have. Moreover, we invited medical staff, renal transplant recipients, and family members to participate in the discussion. This helped establish support for the patients, their relatives, and medical staff. Good supportive relationships can effectively improve the compliance behaviour and self-management abilities of renal transplant recipients [39, 40], thereby gradually improving their ability to manage physical activity, social and psychology function, diet, treatment, and other problems. In addition, when the renal transplant recipients were discharged, a postdischarge plan was made jointly with the recipients and their families so that they were fully prepared for discharge and increased their awareness of self-monitoring and management. The SFA-based nursing intervention provides effective support and a strong guarantee for their disease rehabilitation, psychological rehabilitation, and postoperative adaptation.

## 5. Conclusion

SFA-based nursing interventions can fully tap the strengths of renal transplant recipients and effectively mobilise their enthusiasm, enhance their confidence in overcoming the disease, reduce their anxiety and depression, and improve their overall quality of life. This provides a new idea for the nursing intervention of renal transplant recipients in the future and is of far-reaching significance to promote the maximisation of health outcomes for renal transplant recipients. However, this study has some limitations. This randomised controlled trial was a single-centre study that was only carried out in a tertiary hospital in Zunyi city. Furthermore, because of the time limit, the intervention was implemented only during hospitalisation and the renal transplant recipients were only tracked at one, three, and six months after the intervention. In the future, we aim to expand the sample size, prolong the research cycle, further verify the medium-term and long-term effects of SFA-based nursing interventions, and conduct more in-depth exploration in the field of nursing intervention for renal transplant recipients.

## Figures and Tables

**Table 1:** The specific implementation steps of the research and division of labour among team members.

Team members	Division of labour	Time	Content
Kidney transplant nurse	Complete the training of the members of the research group	Before the intervention	Train SFA intervention content, intervention method, and process
	Deliver the intervention program	During the intervention	Deliver the intervention program

Research nurse A	Random grouping	Before the intervention	Conduct the randomisation and place each randomised number in a sealed opaque envelope

Kidney transplant doctor	Determination of the object of study	Admission day	Strict screening of patients according to inclusion and exclusion criteria, acquire informed consent
	Deal with unexpected situations	During the intervention	Deal with unexpected situation of renal transplant recipients during the intervention, ensure the safety of patients

Dietitian	Assist the intervention	During the intervention	Assist the intervention, provide advice and guidance on nutrition-related issues during the intervention
Rehabilitation nurse			Assist the intervention, provide consultation and guidance on rehabilitation-related issues during the intervention
Psychological counsellor			Assist the intervention, provide consultation and guidance on psychologically related issues during the intervention

Research nurse B	Data collection	Admission day and 1, 3, and 6 months postoperation	Collect baseline and follow-up data

Intensive care nurse	Quality control	During the intervention	Quality control and overall coordination

**Table 2:** Comparison of baseline data of the two groups of renal transplant recipients.

Items	Intervention group (*n* = 37)	Control group (*n* = 34)	*T* or *χ*^2^	*P* value
Age (years)	38.32 ± 9.83	40.97 ± 10.37	−1.103	0.274
Gender				
Male	23 (62.16)	22 (64.71)	0.049	0.824
Female	14 (37.84)	12 (35.29)		
Marriage			0.049^a^	0.825
Unmarried	5 (13.51)	4 (11.76)		
Married	32 (86.49)	30 (88.24)		
Divorced	0 (0.00)	0 (0.00)		
Widowed	0 (0.00)	0 (0.00)		
Education degree			3.026^a^	0.390
Primary school and below	7 (18.92)	2 (5.89)		
Junior middle school	10 (27.02)	13(38.24)		
High school or junior college	7 (18.92)	7 (20.58)		
Bachelor degree or above	13 (35.14)	12 (35.29)		
Dialysis method			1.254^a^	0.685
Haemodialysis	27 (72.97)	28 (82.35)		
Peritoneal dialysis	9 (24.33)	5 (14.71)		
No dialysis	1 (2.70)	1(2.90)		
Duration of dialysis (year)			3.634^a^	0.313
0–1	16 (43.24)	11 (32.36)		
1–3	16 (43.24)	16 (47.05)		
3–5	3 (8.11)	1 (2.94)		
>5	2 (5.41)	6 (17.65)		
BMI (kg/m^2^)	22.00 ± 4.92	22.71 ± 2.29	−0.768	0.445
CCR	7.81 ± 4.11	7.54 ± 2.41	0.329	0.743

*Note*. Data are expressed as *n* (%) or the mean ± standard deviation. ^a^is Fisher's exact probability. BMI: body mass index. CCR: endogenous creatinine clearance.

**Table 3:** Comparison of anxiety and depression scale scores between the two groups of renal transplant recipients.

Items	Group	Baseline	1 month after surgery	3 months after surgery	6 months after surgery	*F*_Time_	*F*_Group_	*F*_Interaction_
HADS	Intervention	11.51 ± 4.29	7.81 ± 3.31	7.29 ± 3.18	7.37 ± 3.25	17.228^*∗∗*^	2.368	1.968
	Control	11.50 ± 3.89	9.85 ± 4.19	8.89 ± 4.25	8.58 ± 3.90			
	*t*	0.014	−2.042^*∗*^	−1.144	−1.210			
	*P*	0.989	0.025	0.202	0.159			

HADS (A)	Intervention	6.37 ± 2.54	4.21 ± 1.85	3.94 ± 1.97	3.91 ± 2.05	16.346^*∗*^	2.875	2.461
	Control	6.44 ± 2.43	5.50 ± 2.44	4.79 ± 2.28	4.52 ± 2.14			
	*t*	−0.063	−1.284^*∗*^	−0.848	−0.610			
	*P*	0.916	0.015	0.097	0.226			

HADS (D)	Intervention	5.13 ± 2.41	3.59 ± 2.14	3.35 ± 1.79	3.45 ± 1.70	9.090^*∗∗*^	1.508^*∗*^	0.869
	Control	5.05 ± 2.53	4.35 ± 2.65	4.11 ± 2.71	4.05 ± 2.69			
	*t*	0.076	−0.758	−0.766	−0.599			
	*P*	0.897	0.187	0.162	0.263			

*Note*. Data are expressed as the mean ± standard deviation. HADS is the Hospital Anxiety and Depression Scale, HADS (A) is the anxiety subscale, and HADS (D) is the depression subscale. ^*∗*^*P* < 0.05, ^*∗∗*^*P* < 0.01.

**Table 4:** Analysis of the generalized estimation equation of anxiety and depression in the two groups at different time points.

	Parameters	*B*	OR value (95% CI)	*P* value	*F*_Time_	*F*_Group_	*F*_Interaction_
Anxiety	(Intercept)	1.265	3.54 (1.08–3.05)	<0.001	21.703^*∗∗*^	4.078^*∗*^	6.149
Group	Intervention	0.141	1.15 (0.93–1.12)	0.203			
	Control	0	1				
	1 month after surgery	−0.059	0.94 (0.79–1.10)	0.525		3.724^*∗*^	
	3 months after surgery	−0.088	0.92 (0.76–1.10)	0.360		1.447	
	6 months after surgery	−0.118	0.89 (0.74–1.10)	0.195		1.706	
	Baseline	0	1			1.617	
Depression	(Intercept)	−0.762	0.47 (0.25–0.88)	0.019	6.956	6.001^*∗*^	9.406^*∗*^
Group	Intervention	−1.053	0.35 (0.15–0.80)	0.012			
	Control	0	1				
	1 month after surgery	−0.859	0.42 (0.17–1.03)	0.058		2.649	
	3 months after surgery	−0.596	0.55 (0.24–1.25)	0.153		8.778^*∗*^	
	6 months after surgery	−0.859	0.42 (0.18–1.02)	0.055		5.796^*∗*^	
	Baseline	0	1			0.143	

*Note*. ^*∗*^*P* < 0.05, ^*∗∗*^*P* < 0.001.

**Table 5:** Comparison of scores of the QOL-RT at different time points between the two groups.

Items	Group	Baseline	1 month after surgery	3 months after surgery	6 months after surgery	*F*_Time_	*F*_Group_	*F*_Interaction_
Physiological function	Intervention	19.40 ± 1.83	21.43 ± 2.19	23.27 ± 1.95	25.59 ± 1.42	284.250^*∗∗*^	5.477^*∗*^	12.789^*∗∗*^
	Control	19.35 ± 2.13	20.05 ± 2.94	21.79 ± 2.56	24.20 ± 2.15			
	*t*	0.052	1.374^*∗*^	1.476^*∗*^	1.389^*∗*^			
	*P*	0.912	0.028	0.008	0.002			

Psychological function	Intervention	20.48 ± 1.81	23.35 ± 3.00	25.00 ± 1.88	24.94 ± 2.12	155.615^*∗∗*^	5.821^*∗*^	6.677^*∗*^
	Control	20.41 ± 1.59	21.64 ± 1.75	23.52 ± 2.12	25.62 ± 1.45			
	*t*	0.075	1.704^*∗*^	1.471^*∗*^	0.680			
	*P*	0.855	0.005	0.003	0.107			

Social function	Intervention	32.78 ± 2.42	39.40 ± 5.72	43.86 ± 5.05	46.89 ± 4.26	202.090^*∗∗*^	4.654^*∗*^	2.217
	Control	32.47 ± 3.57	37.08 ± 6.67	40.52 ± 6.15	44.17 ± 5.52			
	*t*	0.313	2.317	3.335^*∗*^	2.715^*∗*^			
	*P*	0.665	0.120	0.015	0.023			

Treatment	Intervention	19.72 ± 1.66	21.59 ± 1.72	22.72 ± 1.60	23.56 ± 1.84	175.725^*∗∗*^	5.254^*∗*^	9.839^*∗∗*^
	Control	19.64 ± 1.66	20.41 ± 2.03	21.47 ± 1.69	22.61 ± 1.84			
	*t*	0.083	1.183^*∗*^	1.259^*∗*^	0.950^*∗*^			
	*P*	0.835	0.010	0.002	0.034			

The overall quality of life dimension	Intervention	19.67 ± 1.70	20.75 ± 1.62	22.16 ± 1.28	23.21 ± 1.25	115.244^*∗∗*^	1.962	1.900
	Control	19.67 ± 1.38	20.29 ± 1.38	21.55 ± 1.21	22.67 ± 1.27			
	*t*	0.001	0.463	0.603^*∗*^	0.540			
	*P*	0.998	0.202	0.046	0.076			

QOL-RT	Intervention	112.08 ± 6.34	126.54 ± 9.62	137.02 ± 7.69	144.89 ± 7.53	572.118^*∗∗*^	8.283^*∗*^	7.886^*∗∗*^
	Control	111.55 ± 7.43	119.50 ± 11.58	128.8 ± 10.80	138.61 ± 9.09			
	*t*	0.522	7.041^*∗*^	8.145^*∗*^	6.274^*∗*^			
	*P*	0.751	0.007	0.000	0.002			

*Note*. ^*∗*^*P* < 0.05, ^*∗∗*^*P* < 0.01.

## Data Availability

The original data of this study can be obtained through the author's email address 405080390@qq.com.
